# Essential oils from two *Eucalyptus* from Tunisia and their insecticidal action on *Orgyia trigotephras* (Lepidotera, Lymantriidae)

**DOI:** 10.1186/0717-6287-47-29

**Published:** 2014-07-02

**Authors:** Badreddine Ben Slimane, Olfa Ezzine, Samir Dhahri, Mohamed Lahbib Ben Jamaa

**Affiliations:** Institut Supérieur des Sciences et Technologies de l’Environnement de Borj-Cédria, B.P. 1003, Hammam-Lif, 2050 Tunisia; Institut National de Recherches en Génie Rural, Eaux et Forêts, Tunis, Tunisia

**Keywords:** *Eucalyptus*, *Orgyia trigotephras*, Essential oils, Insecticides, Insect control

## Abstract

**Background:**

Essential oils extracted from aromatic and medicinal plants have many biological properties and are therefore an alternative to the use of synthetic products. The chemical composition of essential oils from two medicinal plants (*Eucalyptus globulus* and *E. lehmannii*) was determined and, their insecticidal effects on the third and fourth larval stages of *Orgyia trigotephras* were assessed.

**Results:**

Larvae were collected from Jebel Abderrahmane (North-East of Tunisia), conserved in groups of 50/box (21 × 10 × 10 cm) at a temperature of 25°C. Larvae were tested for larvicidal activities of essential oils*.* Each oil was diluted in ethanol (96%) to prepare 3 test solutions (S1 = 0.05%, S2 = 0.10% and S3 = 0.50%). Essential oils were used for contact, ingestion and Olfactory actions and compared to reference products (*Bacillus thuringiensis* and Decis). Olfactory action of essential oils shows that larvae mortality is higher than contact action, lower than ingestion action. MTM and FTM of S3 of *E. lehmannii* were respectively 1 h 32 min and 1 h 39 min are higher than those of *E. globulus* (MTM = 51 min and FTM = 1 h 22 min 34 sec). Contact action of *E. lehmannii* oil shows low insecticidal activity compared to *E. globulus*. MTM are respectively (1 min 52 sec and 1 min 7 sec), FTM are (2 min 38 sec, 1 min 39 sec), are the shortest recorded for S3, on the third stage of larvae. The fourth stage of larvae, MTM are (2 min 20 sec and 2 min 9 sec), FTM are (3 min 25 sec, 3 min 19 sec). Ingestion action of essential oils is longer than the contact action, since the time of death exceeds 60 minutes for all species.

**Conclusion:**

Results shows that essential oils have a toxic action on nerves leading to a disruption of vital system of insects. High toxic properties make these plant-derived compounds suitable for incorporation in integrated pest management programs.

## Background

Acquired resistance and environmental pollution due to repeated applications of persistent synthetic insecticides have created interest in discovering new natural insecticide products [1]. The use of plants with insecticidal activity has several advantages over the use of synthetic products, natural insecticides are obtained from renewable resources and quickly degradable, the development of insect resistance to these substances is slow, the substances do not leave residues in the environment, they are easily obtained by farmers and they cost less to produce [2]. The effects of essential oils on insects have been the subject of several studies. These oils are formed by a complex mixture of volatile constituents originating from the secondary metabolism of plants and are characterized by a strong scent [3]. The components in essential oils vary not only with plant species but also in relation to climate, soil composition, part of the plant and age of the plant. Since many trees were damaged by insects, the search for insecticides and repellents of botanical origin has been driven by the need to find new products that are effective, furthermore safer and cheaper than current products [4]. Additionally, people prefer natural products than synthetics [5]. Many secondary plant metabolites are known for their insecticidal properties, and in many cases plants have a history of use as home remedies to kill or repel insects [6]. In recent decades, research on the interactions between plants and insects has revealed the potential use of plant metabolites or allelochemicals for this purpose [7]. It is known that some chemical constituents of essential oils have insecticidal properties [8]. In some studies, essential oils obtained from commercial sources were used. Specific compounds isolated from plant extracts or essential oils were tested for fumigation purposes [9].

Among essential oils, Eucalyptus oil, in particular, is more useful as it is easily extractable commercially (industrial value) and possesses a wide range of desirable properties worth exploiting for pest management [10, 11]. Previous studies reported the fumigant toxicity of essential oils from various Eucalyptus species against different developmental stages [12]; furthermore the presence of volatile monoterpenes provides an important defense strategy to the plants, particularly against herbivorous insect pests and pathogenic fungi [13].

This study aims to evaluate toxic activities of essential oils obtained from two *Eucalyptus* species: *Eucalyptus lehmannii* and *Eucalyptus globulus* against third and fourth larval stage of *Orgyia trigotephras.*

## Results

### Essential oils composition

Essential oils efficiency from *E. globulus* and *E. lehmannii* leaves is above 1%. R = 1.25% for *E. globulus* and R = 1.05% for *E. lehmannii*.

As for essential identification, GC and GC/MS analysis of *E. globulus and E. lehmannii* essential oils led to the identification of 32 compounds. The *E. globulus* essential oil profile is characterized by α-pinene (13.61%) and 1.8-cineole (43.18%) as major compounds. Furthermore, *E. lehmannii* is characterized by 1.8-cineole (50.20%) and α-pinene (18.71%) as major compounds. Among other components, the majority belongs to sesquiterpenoïd hydrocarbon volatile compounds (Table 1).

**Table 1:** **Chemical composition (%) of the essential oils of the analyzed** ***Eucalyptus sp*** **.**

RT (min)	Compounds	***E. globulus***	***E. lehmani***
7.40	*α*-Pinene	13.61	18.71
8.94	*β*-Pinene	0.74	0.25
9.50	*β*-Myrcene	0.00	0.21
10.02	*α*-Phellandrene	0.22	0.22
10.87	*β*-Cymene	3.95	0.00
11.21	1. 8-Cineol	43.18	50.20
12.16	*γ*-Terpinene	0.36	2.49
13.31	*α*-Terpinolene	0.50	0.47
14.29	*endo*-Fenchol	0.00	0.41
14.77	*α*-Campholenal	0.20	0.19
15.28	*trans*-Pinocarveol	3.76	2.46
15.45	Camphor	0.00	1.68
16.16	Pinocarvone	2.99	0.60
16.29	Borneol	0.00	0.76
16.71	1-Terpinen-4-ol	0.40	0.57
17.05	*p*-Cymen-8-ol	0.23	0.00
17.24	*α*-Terpineol	1.65	0.00
18.27	Carveol	0.44	0.00
19.14	Carvone	0.22	0.00
22.55	Pulegone	0.15	0.00
17.25	*β*-Fenchol	0.00	2.86
20.63	Bornyl acetate	0.00	0.16
22.83	4-Carene	6.90	9.49
24.80	*α*-Gurjunene	1.33	0.20
25.12	*β*-Caryophyllene	0.81	0.84
25.52	Aristolene	0.70	0.00
25.79	Aromadendrene	10.09	4.26
25.88	*β*-Selinene	0.30	0.00
26.20	*α*-Humulene	0.52	0.00
26.44	Allo-Aromandrene	2.23	1.04
27.22	*α*-Selinene	0.00	0.32
27.50	Ledene	1.06	0.00
Total identified (%)		96.54	98.39

### Insecticidal activity

The evaluation of the contact action of essential oils on larvae of *O. trigotephras* showed a similar effect for the two tested oils. For all concentrations, the MTM and the FTM of larvae treated with essential oils were very short compared to the time of death of larvae treated with Decis.

Ethanol used as a solvent for essential oils, produce no toxic effect on larvae. Oils are revealed to be highly toxic on the third instar larvae. The MTM and FTM are the shortest recorded for a concentration of 0.5 ml. However, *E. lehmannii* oil shows low insecticidal activity compared to the oil of *E. globulus*.

**Figure 1:**
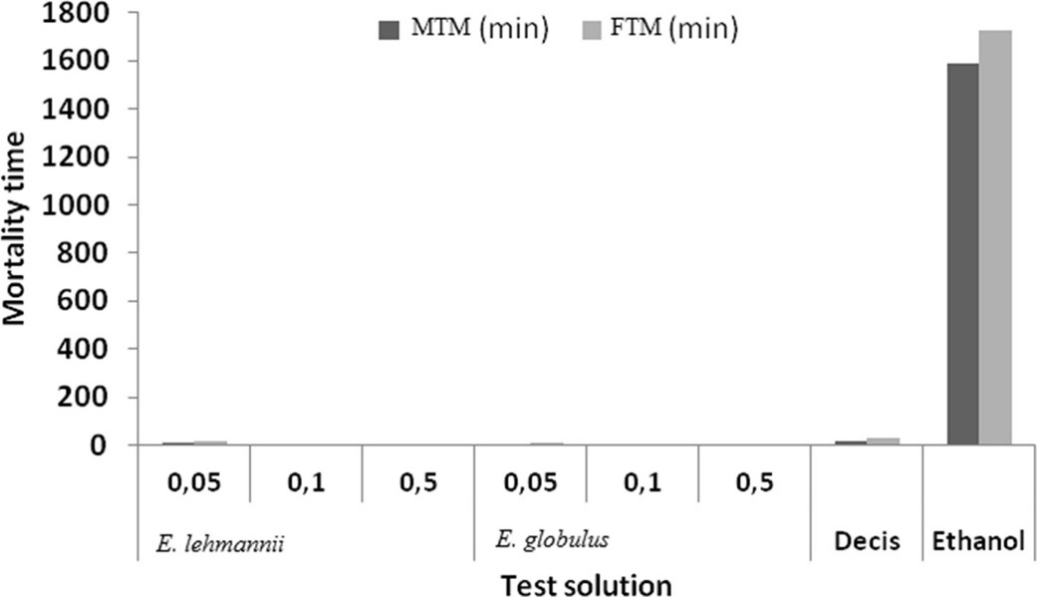
**Mean Time mortality (MTM and FTM) of caterpillars (stage 3), contact processed with essential oils of both species at different concentrations.**

Third instar larvae treated by *E. lehmannii* present a MTM = 11 min 22 sec and FTM = 16 min 55 sec, higher than *E. globulus* (MTM = 2 min and FTM = 5 min 20 sec) for S1 = 0.05%. MTM and FTM, obtained after treatment with 0.5% of *E. lehmannii* were respectively 1 min 52 sec and 2 min 38 sec are higher than those of *E. globulus* (with MTM = 1 min 7 sec and FTM = 1 min 39 sec) (Figure 1).

**Figure 2:**
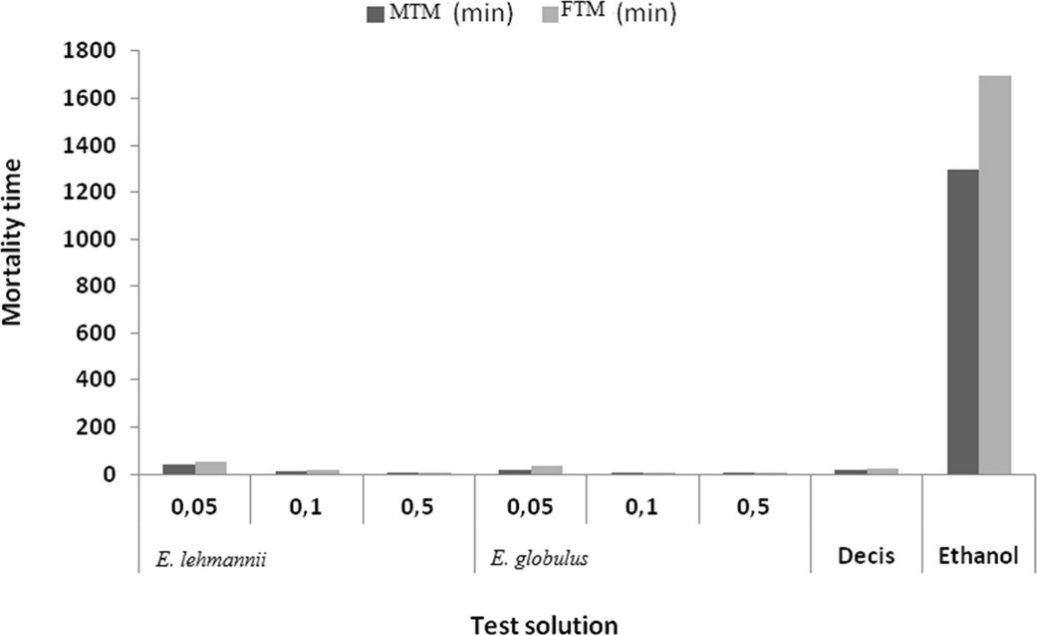
**Mean Time mortality (MTM and FTM) of caterpillars (stage 4) contact processed with essential oils of both species at different concentrations.**

Fourth instar larvae treated by *E. lehmannii* present a MTM = 40 min and FTM = 54 min 49 sec, higher than *E. globulus* (MTM = 16 min 49 sec min and FTM = 32 min) for S1 = 0.05%. MTM and FTM, obtained after treatment with 0.5 ml of *E. lehmannii* were respectively 2 min 20 sec and 3 min 25 sec are higher than those of *E. globulus* (with MTM = 2 min 9 sec and FTM = 3 min 19 sec) (Figure 2).

**Figure 3:**
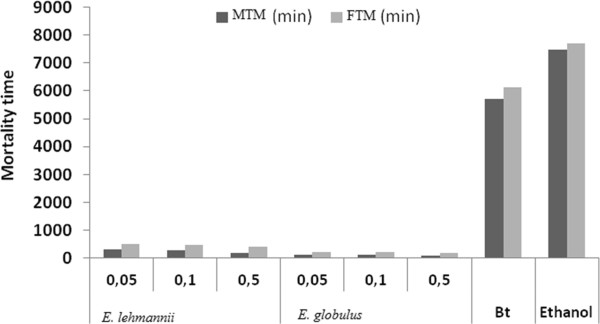
**Mean Time mortality of caterpillars (stage 3) orally processed with essential oils of both species at different concentrations.**

**Figure 4:**
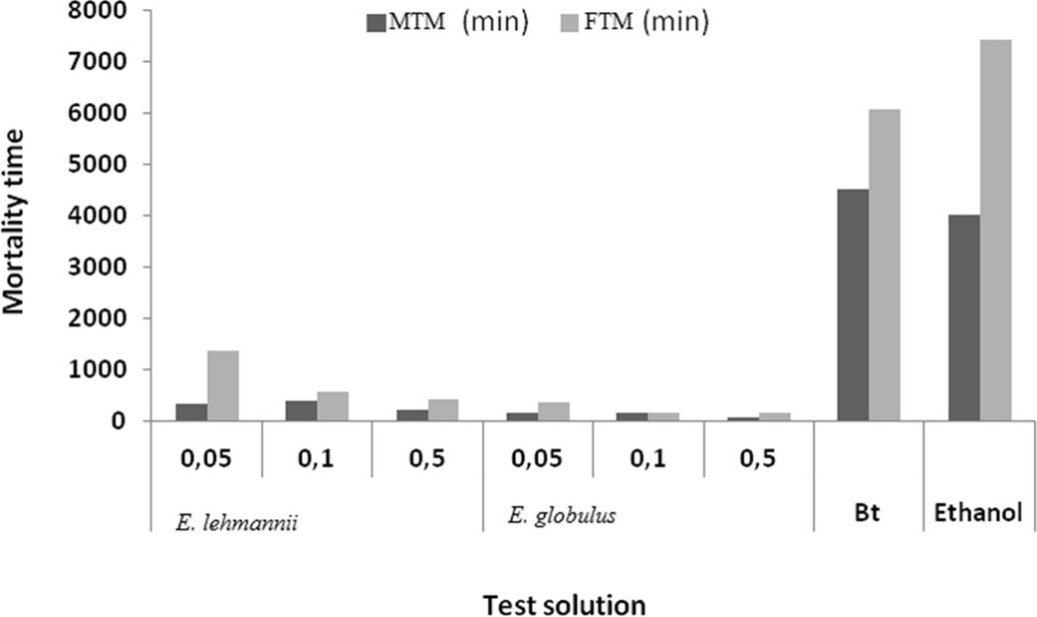
**Mean Time mortality of caterpillars (stage 4) orally processed with essential oils of both species at different concentrations.**

Ingestion action of essential oils is longer than the contact action, since the time of death exceeds 60 minutes for all species. *E. globules* present the best insecticidal effect. Toxicity of *E. globulus* observed for 3 tested concentrations was particularly important when the concentration is high (S3). MTM = 1 h 40 min and FTM = 3 h 4 min for the third instar larvae and MTM = 1 h 37 min and FTM = 3 h 3 min (Figures 3 and 4).

Thus, it is necessary to do insect histology after treatment with oils to detect tissue target and to identify alteration type. Moreover, essential oils can cause cytoplasm coagulation, damage lipids and proteins or cause cell lysis [3]. A similar phenomenon was observed with *B. thurengiensis* treatment.

**Figure 5:**
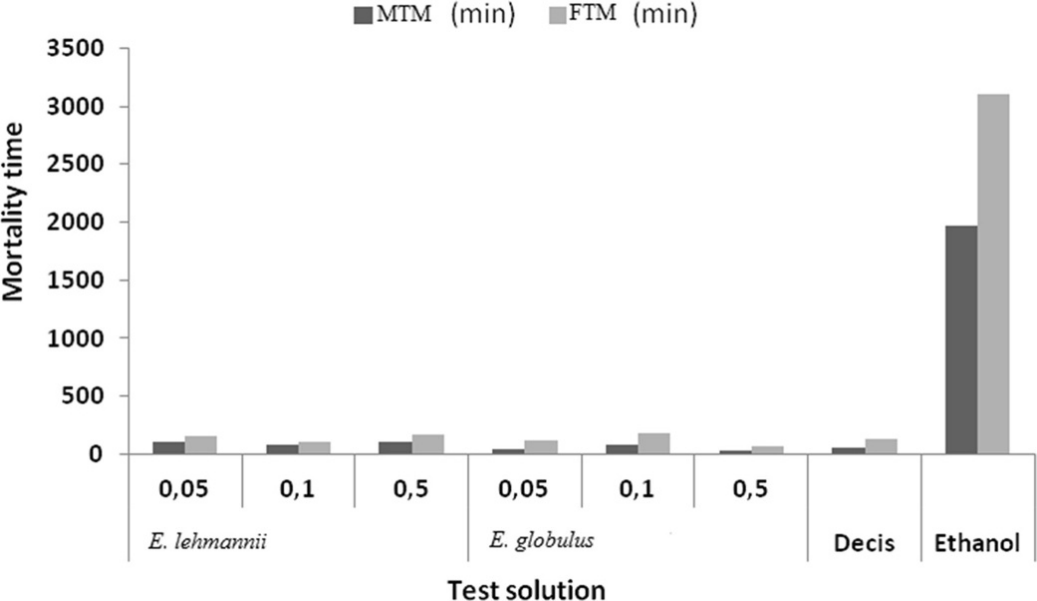
**Mean Time mortality (MTM and FTM) of caterpillars (stage 3) olfactory processed with essential oils of both species at different concentrations.**

As for olfactory action, the third stage of larvae of *O. trigotephras* treated by *E. lehmannii* present a TMM = 4 h 1 min and TFM = 8 h 2 min, higher than *E. globulus* (MTM = 1 h 10 min and FTM = 2 h 11 min) for S1 = 0.05%. MTM and FTM, obtained after treatment with 0.5 ml of *E. lehmannii* were respectively 1 h 32 min and 1 h 39 min are higher than those of *E. globulus* (with MTM = 51 min and FTM = 1 h 22 min 34 sec) (Figure 5).

**Figure 6:**
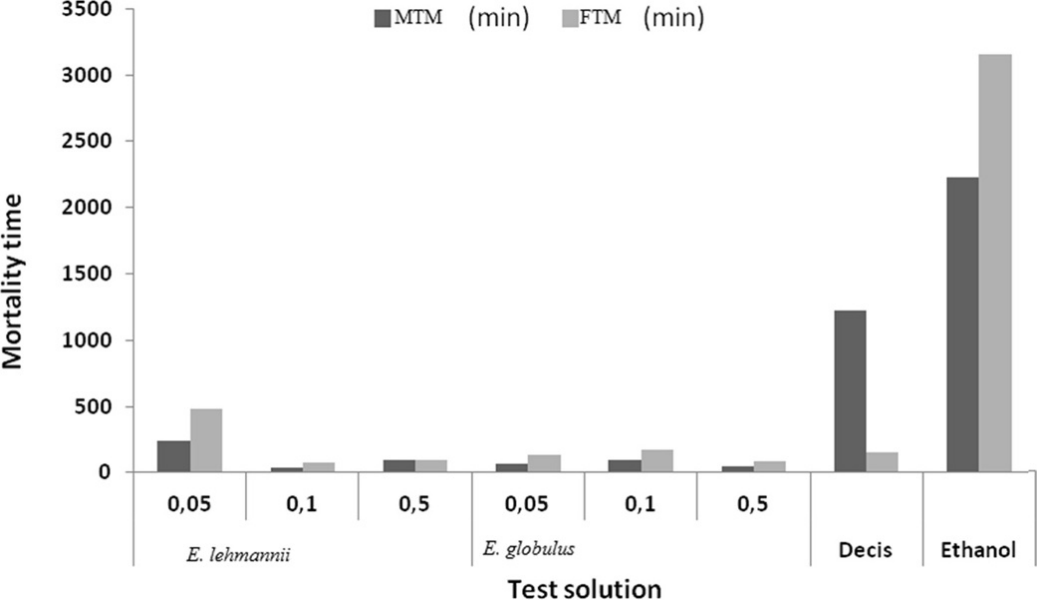
**Mean Time mortality (MTM and FTM) of caterpillars (stage 4) olfactory processed with essential oils of both species at different concentrations.**

The fourth stage of larvae of *O. trigotephras* treated by *E. lehmannii* present a MTM = 1 h 40 min and FTM = 2 h 33 min, higher than *E. globulus* (MTM = 48 min min and FTM = 1 h 56 min) for S1 = 0.05%. MTM and FTM, obtained after treatment with 0.5 ml of *E. lehmannii* were respectively 1 h 50 min and 2 h 54 min are higher than those of *E. globulus* (with MTM = 35 min and FTM = 1 h 2 min) (Figure 6).

Olfactory action of essential oils shows that larvae mortality is higher than contact action, lower than ingestion action. MTM and FTM of *E. lehmannii* are lowest for S3. Larvae mortality is highest for S1 and S2 (Figures 5 and 6). Ingestion action is more effective and contact action is the less effective. It seems that contact action reduce processing times. However, the ingestion action is the most solicit because it indicates the specificity of the product to the insect. Although, more the necessary time for insect mortality is long, it is sure that the product will be toxic to the pest.

## Discussion

Contact action of essential oils is comparable to chemical insecticide that affects the nervous system of larvae as cited by Enan [14] and Cetin et al. [15]. Essential oils have a toxic action on nerves leading to a disruption of vital system of insects [14, 15]. However the highest MTM and FTM of Decis compared to essential oils may be attributed to the limited distribution on the body of larvae unlike essential oils that spread quickly and easily on the back of the insect. The variations between death times resulted from the change in percentages of essential oils components as elucidated by Aslan et al. [16]. The toxicity of certain compounds of essential oils on the fourth instars larvae of *Thaumetopoea pityocampa* has been carried out by Kanat and Alma [17], he showed that turpentine of *Pinus brutia* had the best insecticidal activity (MTM = 0.51 min) due to camphene presence. Kanat and Alma [18] revealed that essential oils of *Thymus vulgaris* composed by carvacrol, *p*-cymene; thymol and *Juniperus communis* composed by camphene and α-pinene have a better insecticide effect than *Lavandula angustifolia* composed by linalool acetate, linalyl, 1,8-cineole and borneol.

*Cymbopogon citratus, Lippia sidoides, Ocimum americanum* and *Ocimum gratissimum* essential oils showed good insecticidal activity against *Aedes aegypti*. Constituents of these oils are the monoterpenoids geranial: citral for *C. citratus*, thymol for *L. sidoides*, E-methylcinnamate for *O. americanum* and eugenol, 1,8-cineole for *O. gratissimum* [19]. *Myroxylum balsamum* essential oil presented good larvicidal activity against *A. aegypti* larvae, the monoterpenes α-pinene and β-pinene were the main constituents [17]. All these constituents are similar as *E. lehmannii* and *E. globules* essential oils.

Treatment with *Bacillus thurengiensis* is long since the action occurs after being gulped, release of toxin and its binding to specific receptors in the midgut of the insect. Ingestion action of *E. globulus* and *E. lehmannii* oils is faster for S1 for the third instar larvae (MTM = 5 h 3 min and FTM = 8 h 33 min) and for the fourth instar larvae (MTM = 5 h 37 min and FTM = 23 h 8 min), than *B. thurengiensis* (MTM = 1j 34 h 57 sec, FTM = 1j 42 h 3 min for the 3^rd^ instar larvae and MTM = 1j 25 h 17 min, FTM = 1j 41 h 14 min for the 4^th^ instar). It is well known that the biopesticide *B. thurengiensis* acts only by ingestion. This bacterium may have a different effect due to the diversity of toxins that can produce [20].

Previous study on monoterpenes’ action on third instar larvae of *Anisakis simplex* Aslan et al. [16] showed that carvacrol was responsible for cells lysis, alteration of the membrane and perforation of the medgut. Essential oil composition varies not only with plant species but also in relation to climate, soil composition, part of the plant and age of the plant [4]. These substances are usually volatile and can be detected by the antennae or tarses of insects. The great majority of the literature on the terpenoids effects on insects has reported growth inhibition, impaired maturation, reduced reproductive capacity, appetite suppression and death of predator insects by starvation or direct toxicity [1]. Monoterpene limonene demonstrated insecticidal activity by penetrating the cuticle of the insect “contact effect”, by respiration “fumigant effect” and through the digestive system “ingestion effect” [21].

Our results clearly demonstrated a high toxic effectiveness relatively to *B. thurengiensis* treatment (ingestion action) and Decis treatement (fumigant and contact action) against both larval phases of this pest.

## Conclusion

To conclude, our study showed that *E. globulus* and *E. lehmannii* essential oils compositions were characterized by the presence of 1.8-cineole (43.18%; 50.20%), α-pinene (13.61%; 18.71%) respectively as major compounds. It is clear that essential oils from *Eucalyptus spp* are rich of monoterpenoid, compounds that possess insecticidal activity against various insect species. High larvicidal properties make derived compounds suitable for incorporation of integrated pest management program. These results show that application of natural plant products as *E. lehmannii* and *E. globules* which have a toxic effect against larvae of *Orgyia trigotephras* can be a potential method in environmental- friendly control management.

## Methods

### Plant material and larvae collect

Our study was carried out in the arboretum of Jebel Abderrahmaen (North-east of Tunisia). Four branches from the quadrant (N, S, E and W) were cut off from five trees of the two *Eucalyptus* species; *E. lehmannii* and *E. globules* using a telescopic tree pruner. Branches were separately placed in plastic bags. In the lab, leaves were carried out and air-dried at room temperature (20-25°C) for one week and stored for essential oil extraction. Larvae were collected from Jebel Abderrahmane, conserved in groups of 50 per box (21 × 10 × 10 cm) at a temperature of 25°C and fed every two days on fresh leaves of *Erica multiflora* Third and fourth stage larvae of *Orgyia trigotephras* were tested for larvicidal activities of essential oils*.*

### Essential oil extraction and volatile compounds identification

100 g of dry matter of leaves were used for Essential oils extraction by hydro-distillation method during 90 min using a modified Clevenger-type apparatus. Anhydrous sodium sulphate was used to remove water after extraction. The extracted oils were stored in Eppendorf safe-lock tubes and stored at −4°C.

Essential oils were analyzed by gas chromatography (GC) using a Hewlett-Packard 6890 gas chromatograph (Agilent Technologies, Palo Alto, California, USA) equipped with a flame ionization detector (FID) and an electronic pressure control (EPC) injector. A polar HP-Innowax (PEG) column (30 m × 0.25 mm, 0.25 mm film thickness) and an apolar HP-5 column (30 m × 0.25 mm coated with 5% phenyl methyl silicone, and 95% dimethyl polysiloxane, 0.25 mm film thickness) from Agilent were used. Carrier gas flow (N2) was 1.6 ml/min and the split ratio 60:1. Analyses were performed using the following temperature program: oven kept isothermally at 35°C for 10 min, increased from 35 to 205°C at the rate of 3°C/min and kept isothermally at 205°C for 10 min. Injector and detector of temperatures were held, respectively, at 250 and 300°C. The GC/MS analyses were made using an HP-5972 mass spectrometer with electron impact ionization (70 eV) coupled with an HP-5890 series II gas chromatograph. An HP-5MS capillary column (30 m × 0.25 mm coated with 5% phenyl methyl silicone, and 95% dimethyl polysiloxane, 0.25 μm film thicknesses) was used. The oven temperature was programmed to rise from 50 to 240°C at a rate of 5°C/min. The transfer line temperature was 250°C. Helium was used as carrier gas with a flow rate of 1.2 ml/min and a split ratio of 60:1. Scan time and mass range were 1 s and 40e300 m/z respectively.

Essential oil volatile compounds were identified by calculating their retention index (RI) relative to (C9-C18) n-alkanes (Analytical reagents, Labscan, Ltd, Dublin, Ireland) and data for authentic compounds available in the literature and in our data bank, and also by matching their mass spectrum fragmentation patterns with corresponding data stored in the mass spectra library of the GC-MS data system (NIST) and other published mass spectra [22]. The relative percentage amount of each identified compound was obtained from the electronic integration of its FID peak area.

### Essential oils efficiency

Essential oils efficiency (R) is expressed by the ratio between the amount of oil extracted and the amount of plant material used for extraction. R (%) = (mass of the essential oil obtained per mass of plant material used)*100.

### Preparation of test solutions and chemical insecticide

Each oil was diluted in ethanol (96%) to prepare 3 test solutions (S1 = 0.05%, S2 = 0.10% and S3 = 0.50%). The essential oils were tested by contact action, ingestion action and olfactory action. The larvicidal effect of essential oils by contact is appreciated by comparison to a chemical insecticide Delta-metrine “Decis” (Atlas Agro-Tunisia). Ethanol used for dilutions was already used as control. The larvicidal effect by ingestion of essential oils is assessed by comparison to a biological insecticide *Bacillus thuringiensis* (reference product, provided by Atlas Agro-Tunisia).

### Larvae preparation

Ten larvae were placed in Petri dishes (R = 9 cm). This experiment was replicated 6 times for each test. The rest of the larvae were placed in plastic boxes.

### Contact and ingestion action of essential oils

Firstly, 10 μl of each oil solution prepared was deposited on the back of each larva; a total of 60 larvae from 3^rd^ and 4^th^ stage were used and secondary 100 μl from each oil concentration are spread over *Erica multiflora* leaves. Leaves are left in open air until total absorption, than are placed in Petri dishes with 10 fasted larvae to test ingestion action.

### Residual toxicity test and evaluation of the insecticidal

100 μl of each test solution were deposited on the bottom of Petri dishes (R = 9 cm) and dried for 20 min at 21°C, 10 larvae per replication were placed to test olfactory action.

The larvicidal activity of essential oils, reference products (*Bacillus thuringiensis*, Decis) and ethanol were determined by measuring the average time of mortality rate (MTM) corresponding to the time required to kill 50% of larvae and the final time of mortality (FTM) corresponding to the death of the total larvae.

### Statistical analysis

The statistical treatment of data is performed using SPSS (Version 10.0). MTM and FTM were analyzed for variance by the Fisher test to test the hypothesis of equality of means at the threshold 5%. It is complemented by multiple comparisons of means by the LSD test (Least Significant Difference).
